# Evaluation of the Bond Strength of Polyetherketoneketone to Dental Ceramic

**DOI:** 10.3290/j.jad.b3710327

**Published:** 2022-12-23

**Authors:** Bahar Tekli, Gulfem Ergun

**Affiliations:** a Research Assistant, Department of Prosthodontics, Faculty of Dentistry, Gazi University, Ankara, Turkey. Idea, hypothesis, performed experiments, wrote manuscript.; b Professor, Department of Prosthodontics, Faculty of Dentistry, Gazi University, Ankara, Turkey. Experimental design, proofread the manuscript, contributed substantially to discussion.

**Keywords:** PEKK, ceramic, surface roughness, shear bond strength.

## Abstract

**Purpose:**

To evaluate the efficacy of different surface treatments on the bond strength of polyetherketoneketone (PEKK) to ceramic materials.

**Materials and Methods:**

PEKK test specimens were separated into four groups according to surface treatments (group S: sandblasting; group A: acid etching; group SA: sandblasting + acid etching; group C: control). Surface roughness values of PEKK specimens were measured before and after surface treatment. After the prepared PEKK specimens were bonded to lithium-disilicate glass-ceramic specimens with resin cement, they were kept in distilled water at 37°C for 24 h. Half of the specimens in each surface treatment group were thermocycled. All test specimens underwent shear bond strength testing. Failure modes were assessed using light microscopy and SEM.

**Results:**

Statistical analysis demonstrated that surface treatments created significant roughness on the PEKK surfaces (p = 0.005). While group S showed the highest roughness values, group A showed the lowest. Of the thermocycled test groups, the sandblasting + acid etching group obtained the highest shear bond strengths. Among the non-thermocycled test groups, the sandblasting surface treatment group achieved the highest shear bond strengths.

**Conclusions:**

The application of surface treatments to enhance the bond strength of PEKK to ceramics has proven to be effective.

The use of computer-aided design/computer-aided manufacturing (CAD/CAM) has increased thanks to the rapid development of digital technologies in dentistry. As an alternative to conventional methods, CAD/CAM systems are used in the production of dental restorations, as they offer a better quality and are more precise and efficient.^37^ Today, many CAD/CAM materials (ceramics, metals, and polymers) that can be used in the digital production process are used in dentistry.^12,13,43^

The poor esthetic appearance of porcelain-fused-to-metal, the difficulty in providing marginal fit, increased weight of the prosthesis, metallic taste, and allergic reactions to metals have increased the interest in alternative framework materials.^21^ Dental ceramics are considered best able to mimic natural dentition and provide optimum esthetics.^51^

Today, polymers are fracture-resistant, inert, biocompatible, and economical dental materials with lower weight and lower density than metals.^41^ Polyetheretherketone (PEEK) and polyetherketoneketone (PEKK) are thermoplastic polymers belonging to the polyaryletherketone (PAEK) family.^9^ PEKK represents a newer member of the PAEK family. Due to the additional ketone group, PEKK shows better mechanical and physical properties compared to PEEK, such as better polishing, an 80% higher compressive strength, and a bone-like elastic modulus.^9,24^ These two polymers are considered alternative dental framework materials for metal-reinforced ceramics, owing to their adequate fracture strength and better stress distribution and shock absorption. According to the manufacturer, PEKK has a compressive strength (246 MPa) similar to dentin (297 MPa), but a lower elastic modulus (5.1 GPa).^27^ With all these mechanical advantages as well as biocompatibility, PEKK is emerging as a suitable substructure material for fixed prostheses, removable prostheses, and implant-supported prostheses.^28^

However, their grayish-white color and low translucency limit the use of PAEKs as monolithic tooth restoration materials.^28^ To obtain esthetic results, they must be used with a veneer. While a composite resin can be used as a veneer on PEEK and PEKK,^14,24,45,46^ lithium-disilicate glass-ceramic crowns can be bonded via resin cement to the PEKK telescopic substructure.^9^

In general, many properties of the restorative material, such as wettability and friction coefficient, are significantly affected by the surface roughening processes.^30^ To obtain a good bond between PEEK and the veneer, it is necessary to first increase the surface roughness with various treatments. Studies have reported that it is essential to increase bond strength by enabling the resin material to flow into the microretentive areas thus formed.^35,53^ Increasing surface roughness also contributes to micromechanical retention by decreasing surface tension and increasing both hydrophilicity and surface area.^23^ For this reason, the PEEK surface must be treated mechanically or chemically to obtain sufficient bond strength to the veneer.^25^ Surface treatments such as acid etching, acetone, silica coating, sandblasting, plasma, and laser are applied to roughen PEEK surfaces, thereby providing optimal bond strength between the PEEK surface and resin-based materials.^3,6,11,35,38,49,53^

PEKK also has low surface energy, similar to PEEK. However, these polymers have different chemical structures. Many surface roughening processes such as acid etching, silica coating, sandblasting, and plasma have been used to enhance the surface energy of PEKK materials and solve the bonding problem with resin-based materials.^10,14,15,26,28,36,42,48,52^ Several studies have examined the efficacy of different surface treatments on the bond strength between composite resin veneer materials and PEKK.^10,14,28,36,48,52^ However, studies have rarely been conducted regarding the bond strength of PEKK, a new-generation polymer, to ceramics.

In the present study, the efficacy of different surface treatments applied to PEKK in providing sufficient shear bond strength of PEKK to lithium-disilicate glass-ceramics were evaluated. At the same time, the effect of oral conditions on bond strength was observed by imitating clinical conditions through thermocycling. The hypotheses were that the surface treatments would affect the surface roughness values of PEKK; surface treatments would have an effect on the bond strength of PEKK to the ceramic; and thermocycling would have a negative effect on shear bond strength.

## MATERIALS AND METHODS

### Study Materials

The materials used in this study are shown in Table 1.

**Table 1:** Composition of materials used in the study

Material	Product name	Composition	Lot No	Manufacturer
Polyetherketoneketone	Pekkton ivory milling blank	Polyetherketoneketone, titanium dioxide	211145	Cendres+Metaux; Biel/Bienne, Switzerland
Surface treatment	Piranha solution	H_2_SO_4_ (98%):H_2_O_2_ (30%) = 10:3	7000899914	Albar Kimya; Kocaeli, Turkey
Surface treatment	Aluminum oxide	110 μm particle size aluminum oxide (Al_2_O_3_ 99.6%)	1201204	Dentona dento-blast; Dortmund, Germany
Adhesive agent	Pekk Bond	MMA, diphenyl (2,4,6-trimethylbenzoyl) phosphine oxide, activators, stabilizers	2019011494	Anaxdent; Stuttgart, Germany
Lithium-disilicate glass-ceramic (CAD/CAM block)	IPS e.max CAD, HT A2 C14	SiO_2_ 57–80%, Li_2_O 11–19%, K_2_O 0–13%, P_2_O_5_ 0–11%, ZrO_2_ 0–8%, ZnO 0–8%, others and coloring oxides 0–12%	Y18855	Ivoclar Vivadent; Schann, Liechtenstein
Ceramic etching	Ultradent Porcelain Etch	9% hydrofluoric acid	BJ7V1	Ultradent; South Jordan, UT, USA
Ceramic primer	G-Multi PRIMER	Ethanol, MDP, MDTP, γ-methacryloxypropyl trimethoxysilane (silane), methacrylate monomer	1911081	GC; Tokyo, Japan
Resin cement	G-CEM LinkForce, (A2)	Paste A: bis-GMA, UDMA, barium glass, initiator, pigments Paste B: bis-MEPP, UDMA, dimethacrylate, barium glass, initiator	1911011	GC
Autopolymerizing acrylic resin	Integra Orthodontic Acrylic	MMA 95%, EDMA 5%	190921	Birleşik Grup Dental; Ankara, Turkey

PEKK: polyetherketoneketone; H_2_SO_4_: sulfuric acid; H_2_O_2_: hydrogen peroxide; MMA: methylmethacrylate; 10-MDP: 10-methacryloyloxydecyl dihydrogen phosphate; MDTP: methacryloyloxidecyl dihydrogen thiophosphate; bis-GMA: bisphenol A glycidyl methacrylate; bis-MEPP: 2,2-bis(4-methacryloxypolyethoxyphenyl) propane; UDMA: urethanedimethacrylate; EDMA: ethyleneglycol dimethylacrylate; SiO_2_: silicon dioxide; Li_2_O: lithium oxide; K_2_O: potassium oxide; P_2_O_5_: phosphorus pentoxide; ZrO_2_: zirconium dioxide; ZnO: zinc oxide.

### Specimen Preparation

For this in-vitro study, 88 PEKK test specimens (dimensions: 16 x 16 x 2 mm) from CAD/CAM blanks (Pekkton ivory, Cendres+ Métaux; Biel/Bienne, Switzerland) were fabricated and embedded in autopolymerizing acrylic resin (Integra Orthodontic Acrylic, Birleşik Grup Dental; Ankara, Turkey) in a polyethylene mold. The test specimens were polished sequentially using 600-, 800-, and 1200-grit silicon carbide papers (English Abrasives; London, UK) for 60 s under 30 N pressure with a polishing machine (Metkon Gripo 2V Grinder-Polisher; Bursa, Turkey) under continuous water cooling. After polishing, the test specimens were placed in distilled water for 5 min in an ultrasonic cleaning device (BioSonic UC1, Coltène/Whaledent; Altstätten, Switzerland) and air dried before surface treatment procedures.

80 ceramic test specimens (dimensions: 12 x 12 x 2 mm) were prepared from IPS e.max CAD HT A2 C14 (Ivoclar Vivadent; Schaan, Liechtenstein) lithium-disilicate glass-ceramic blocks using a precision cutting device (Microcut 201, Metkon; Bursa, Turkey) and a diamond cutting disk (IsoMet Diamond Wafering Blades 15LC, 11- 4255, 127 x 0.4 mm L114255-R3 Buehler; Lake Bluff, IL, USA). The specimens were sintered in a porcelain furnace per manufacturer’s recommendations, and all of them were sandblasted with 110-µm Al_2_O_3_ (Dentona dento-blast; Dortmund, Germany) from a 1-cm distance at a pressure of 2 bars for 60 s and an angle of 90 degrees, to ensure standardization and compliance with clinical-use procedures. The specimens were then cleaned ultrasonically in distilled water for 60 s.

### Surface Treatments

The PEKK test specimens were then separated into 4 surface treatment groups as follows (n = 22 per group): S: sandblasting; A: acid etching; SA: sandblasting + acid etching, and C: control group with no surface treatment. Before the surface treatments were applied, the surface roughness values of the test specimens of all groups were measured and recorded by the same person.

Group S PEKK specimens were sandblasted with 110 µm Al_2_O_3_ 110 µm at 2 bars of pressure at a 1-cm distance and an angle of 90 degrees for 60 s in a sandblasting machine (Basic Eco; Renfert, Germany).

Group A PEKK specimens were treated with 100 μl of piranha solution for 30 s with a micropipette (Thermo Scientific Finnpipette F1; Waltham, MA, USA). The specimens were rinsed with distilled water for 30 s.

Group SA PEKK specimens were sandblasted with 110-µm Al_2_O_3_ 110 µm at 2 bars of pressure at a 1-cm distance and an angle of 90 degrees 90 for 60 s in a sandblasting machine. Sandblasted PEKK surfaces were wiped with alcohol and allowed to dry. 100 μl of piranha solution was applied for 30 s with a micropipette. The specimens were rinsed with distilled water for 30 s.

No surface roughening was performed on the group C PEKK specimens.

### Analysis of Surface Properties

The surface roughness values of 10 PEKK specimens selected randomly from each group (n = 22) were measured with a profilometer (Perthometer M2, Mahr; Gottingen, Germany) before and after surface treatments. Parallel measurements in the horizontal direction were taken from the surface of each PEKK specimen, passing through 3 points determined on the same vertical line. For each group of surface treatments, the average surface roughness (Ra) value (ie, the arithmetic average of the absolute values) was determined.

After surface treatment, the topographies of the PEKK specimens were analyzed by SEM (JEOL, JSM-6060LV, SEM; Tokyo, Japan), selecting 2 specimens per group whose surface roughness values were closest to the group average. The selected specimen surfaces were dried and sputter-coated with a gold-palladium film with 10 mA current, and 2 mbar/Pa combustion chamber pressure for 165 s in a gold-palladium sputter-coater (Sputter Coater SC502, Polaron, VG Microtech; Uckfield, UK). SEM images were obtained at 250X, 500X, 1000X and 2500X magnification.

### Adhesion of PEKK to Lithium-Disilicate Glass-Ceramic

The surfaces of PEKK specimens were wiped with alcohol and allowed to dry. A thin layer of adhesive Pekk Bond (Anaxdent; Stuttgart, Germany) was applied using a brush, as per the manufacturer’s instructions, onto bonding surfaces delimited by 8-mm-diameter holes in 0.10-mm-thick pieces of Teflon tape. Polymerization was performed for 90 s with a laboratory-use light-polymerization device (Anaxdent Light Box; Stuttgart, Germany) with a wavelength range 380–550 nm.

Lithium-disilicate glass-ceramic specimens were cleaned by wiping with alcohol, then letting the surfaces dry. Then, the ceramic preparation protocol was used, consisting of hydrofluoric acid (Ultradent Porcelain Etch, Ultradent; South Jordan, UT, USA) and a primer containing silane (G-Multi Primer, GC; Tokyo, Japan). The surfaces of the specimens were covered with hydrofluoric acid for 20 s. Then they were rinsed with distilled water for 30 s and dried. A ground-glass image was obtained on their surfaces. Then, a drop of primer was applied in a thin layer to the ceramic surfaces using a brush. The surfaces were gently air dried to evaporate the ethanol solvent in the primer.

**Fig 1:**
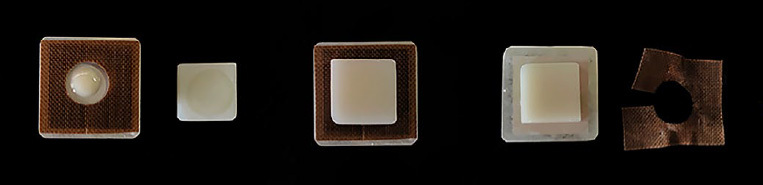
Bonding procedure of PEKK to lithium-disilicate glass-ceramic.

**Fig 2:**
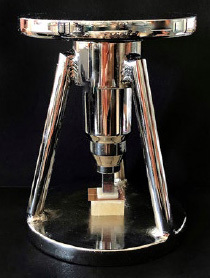
Special loading device that applies a 0.94 kg load to standardize pressure during cementation.

**Fig 3:**
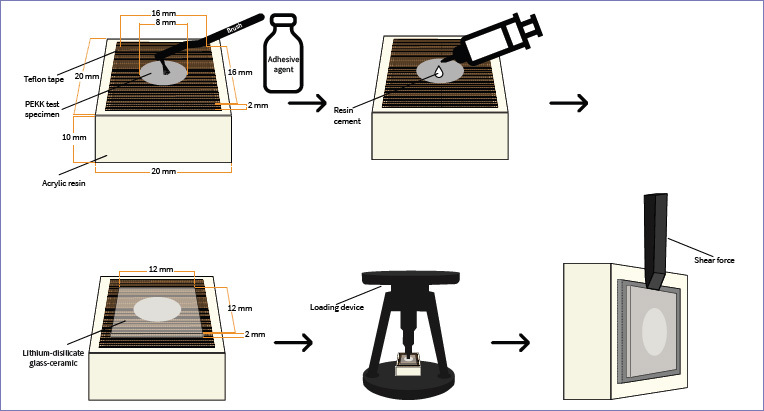
Schematic diagram of bonding PEKK to lithium-disilicate glass-ceramic.

Next, lithium-disilicate glass-ceramic specimens and PEKK specimens were bonded to each other using dual-cure self-adhesive resin cement (G-CEM Link Force, GC; Tokyo, Japan). Resin cement was applied to PEKK specimens, whose bonding surface was delimited by an 8-mm-diameter hole in a 0.10-mm-thick piece of Teflon tape in order to create a standard and homogeneous resin cement thickness (Fig 1). After bonding the PEKK specimens to the lithium-disilicate glass-ceramic specimens, they were placed in a special loading device (OSTIM; Ankara, Turkey) which applied a standard pressure of 0.94 kg during cementation (Fig 2). To ensure that cement excess became rubbery and easy to clean off, the cement was partially polymerized using a curing light (Valo, LED, Ultradent) with an output of 1000 mW/cm^2^ for 2 s on 4 different surfaces. Then, excess cement and the Teflon tape were removed. Polymerization was then performed from 4 different surfaces for 20 s with the LED curing light. The schematic diagram of the bonding procedure of PEKK to lithium-disilicate glass-ceramic is shown in Fig 3.

### Artificial Aging of Test Specimens with Thermocycling

After bonding PEKK to lithium-disilicate glass-ceramic, to complete polymerization, all test specimens were kept in distilled water at 37°C in incubator (Kottermann Labortechnik; Uetze Hänigsen, Germany) for 24 h. All specimens to be subjected to shear bond strength testing were separated into two subgroups: thermocycled and non-thermocycled. In each group, half of the specimens (n = 10) were subjected to thermocycling (TC+) for 6000 cycles (5°C and 55°C; dwell time: 25 s per bath; transfer time: 10 s) in a thermocycling machine (SD Mechatronik Thermocycler FT 200, Julabo; Seelbach, Germany). The other half were kept in distilled water (TC-) at 37°C in an incubator until thermocycling ended.

### Shear Bond Strength Testing

**Fig 4:**
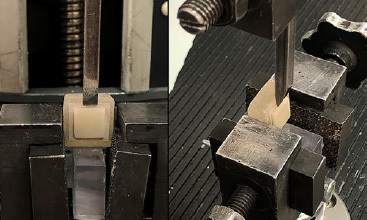
Shear bond strength test.

A universal testing machine (Lloyd-LRX, Lloyd Instruments; Fareham, UK) was used to perform all tests on the specimens. The knife-edge-shaped loading blade was placed parallel to the PEKK/lithium-disilicate glass-ceramic bonded interface. A shear force was applied at a 0.5 mm/min crosshead speed until bonding failure occurred (Fig 4). The maximum load at which the lithium-disilicate glass-ceramic debonded from the PEKK surface was measured in Newtons (N). Shear bond strength was calculated MPa as follows: maximum load (N) ÷ the bonded area of the PEKK-lithium-disilicate glass-ceramic unit (mm^2^).

### Bond Failure Analysis

The PEKK surfaces were examined at 8X magnification under a light microscope (Leica MZ 12, Leica Microsystems; Wetzlar, Germany) to assess the failure mode after debonding. Failure modes were scored as 0, 1, 2, and 3 using the adhesive remnant index (ARI) proposed by Årtun and Bergland (Table 2).^1^ One test specimen from each surface treatment group (S, A, SA, and C) with scores of 0, 1, 2, and 3 were analyzed by SEM.

**Table 2:** Adhesive remnant index (ARI) scores

ARI score	Criteria
0	No resin cement left on the bonding area of PEKK specimen
1	Less than half of the resin cement left on the bonding area of PEKK specimen
2	More than half of the resin cement left on the bonding area of PEKK specimen
3	All resin cement left on the bonding area of the PEKK specimen

### Statistical Analysis

MedCalc Statistical Software version 12.7.7 (MedCalc Software; Ostend, Belgium) was used to analyze the data. To define continuous variables, descriptive statistics were used (mean ± SD, maximum [max], median, minimum [min]). Two variables that were independent and not normally distributed were compared using the Mann-Whitney U-test. The Kruskal-Wallis test was performed to compare more than two variables that were independent and not normally distributed. Dependent and non-normally distributed variables were compared using the Wilcoxon test. Statistical significance was set at 0.05.

## RESULTS

### Surface Properties

The average surface roughness values (Ra) and SD of the PEKK specimens belonging to the 4 different surface treatment groups are shown in Table 3, before (1st measurement) and after (2nd measurement) different surface treatments.

**Table 3:** Surface roughness values

PEKK surface treatment group		Group S (sandblasting)	Group A (acid etching)	Group SA (sandblasting + acid etching)	Group C (control, no treatment)	p^1^
Surface roughness values (Ra, µm) 1st measurement	Mean±SD	0.26±0.01	0.24±0.02	0.23±0.01	0.24±0.02	0.001
	Median (min.-max.)	0.27 (0.24–0.28)	0.24 (0.21–0.28)	0.23 (0.21–0.24)	0.23 (0.21–0.27)	
Surface roughness values (Ra, µm) 2nd measurement	Mean±SD	1.73±0.07	0.42±0.06	0.82±0.05	0.24±0.02	<0.001
	Median (min.-max.)	1.73 (1.62–1.85)	0.41 (0.33–0.52)	0.82 (0.75–0.89)	0.23 (0.21–0.27)	
p^2^		0.005	0.005	0.005	1.000	

Significance level p^1^<0.05, p^2^<0.05; Kruskal-Wallis test^1^, Wilcoxon Signed Rank test^2^.

The differences in surface roughness values (2nd measurement) between the S, A, SA, and C groups were statistically significant (p < 0.001).

The differences between the Ra values before treatment (1st measurement) and after surface treatment (2nd measurement) for groups S, A, and SA were statistically significant (p = 0.005). Accordingly, in the group comparisons, the surface roughness values increased after surface treatment in all surface treatment groups.

Post-hoc pairwise comparison of post-treatment surface roughness in all groups yielded the following results: statistically significant differences were observed between groups S and A, S and SA, S and C, A and SA, A and C, and SA and C (p < 0.001) (Table 4).

**Table 4:** Post-hoc analysis of surface roughness values

PEKK surface treatment groups		p^3^
Group S	Group A	<0.001
	Group SA	<0.001
	Group C	<0.001
Group A	Group SA	<0.001
	Group C	<0.001
Group SA	Group C	<0.001

Significance level p<0.008; Mann-Whitney U-test^3^, Bonferroni correction.

**Fig 5:**
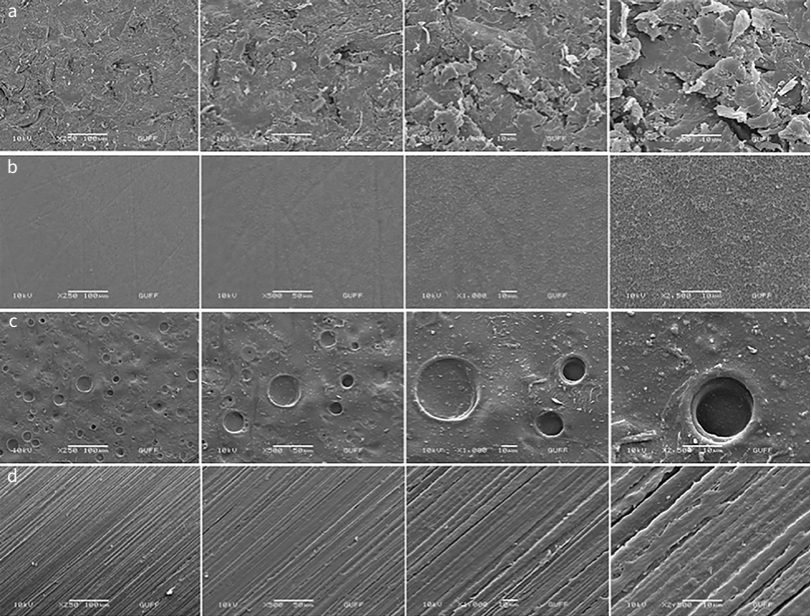
SEM images of PEKK surfaces after different treatments (magnifications 250X, 500X, 1000X, 2500X, left to right). a) Group S; b) Group A; c) Group SA; and d) Group C.

SEM images obtained after the different PEKK surface treatments are shown in Fig 5. In group S, an irregular, cracked surface with peaks, valleys, and embedded polygonal-shaped Al_2_O_3_ particles was observed, while in group A, a large number of round micropores and honeycomb-like surface features were formed. In group SA, a porous structure with small cracks was evident. In group C, although some small scratches, grooves, and small PEKK chips were produced by the silicon carbide abrasive paper used during polishing, a smooth, regular, flat surface was detected.

### Shear Bond Strength and Failure Analysis

The shear bond strengths of all groups (S, A, SA, and C) are shown in Table 5. The differences in shear bond strengths among the thermocycled surface treatment groups (STC+, ATC+, SATC+, and CTC+) were not statistically significant (p = 0.249). However, statistically significant differences (p < 0.001) were found among the non-thermocycled surface treatment groups (STC-, ATC-, SATC-, and CTC-).

**Table 5:** Shear bond strengths

PEKK surface treatment groups		Group S (sandblasting)	Group A (acid etching)	Group SA (sandblasting + acid etching)	Group C (control, no treatment)	p^1^
Thermocycled groups		STC+	ATC+	SATC+	CTC+	
Shear bond strengths (MPa)	Mean±SD	11.02±3.25	10.86±2.95	12.3±3.1	8.41±4.01	0,249
	Median (min.-max.)	9.77 (8.04-18.01)	11.33 (6.21-15.2)	10.88 (9.21-18.49)	9.38 (0.77-13.84)	
Non-thermocycled groups		STC-	ATC-	SATC-	CTC-	
Shear bond strengths (MPa)	Mean±SD	13.53±1.84	11.86±2.83	8.67±2.43	9.14±2.53	<0.001
	Median (min.-max.)	12.77 (11.77-16.65)	12.57 (4.19-14.09)	9.24 (4.83-11.89)	8.64 (6.96-15.24)	
p^2^		0.015	0.393	0.011	0.853	

Thermocycled (TC+); non-thermocycled groups (TC-). Significance level p^1^<0.05, p^2^<0.05; Kruskal-Wallis test^1^, Mann-Whitney U-test^2^.

The shear bond strength of the STC+ subgroup was found to be statistically significant lower (p = 0.015) than that of the STC- subgroup. The shear bond strength of the SATC+ subgroup was higher than that of the SATC- subgroup, and this difference was also statistically significant (p = 0.011). Nevertheless, the difference between the ATC+ and ATC- subgroups (p = 0.393), and between the CTC+ and CTC- subgroups (p = 0.853) was not statistically significant.

Post-hoc pairwise comparisons in the non-thermocycled surface treatment groups (STC-, ATC-, SATC-, and CTC-) showed that the differences in the shear bond strengths between the STC- and SATC- (p < 0.001), STC- and CTC- (p = 0.001), and ATC- and SATC- (p = 0.003) groups were statistically significant, although no statistically significant differences existed between the STC- and ATC- (p = 0.436), ATC- and CTC- (p = 0.019), and SATC- and CTC- (p = 0.971) groups (Table 6).

**Table 6:** Post-hoc analysis of the shear bond strengths of the non-thermocycled groups

		p^2^
STC-	ATC-	0.436
	SATC-	<0.001
	CTC-	0.001
ATC-	SATC-	0.003
	CTC-	0.019
SATC-	CTC-	0.971

Significance level p^2^<0.008; Mann-Whitney U-test^2^, Bonferroni correction.

In the post-hoc pairwise comparison between the shear bond strengths of the STC+, ATC+, SATC+, and CTC+ groups, significant differences were not found between any two groups (Table 7).

**Table 7:** Post-hoc analysis of the shear bond strengths of the thermocycled groups

		p^2^
STC+	ATC+	0.853
	SATC+	0.143
	CTC+	0.393
ATC+	SATC+	0.579
	CTC+	0.165
SATC+	CTC+	0.105

Significance level p^2^<0.008; Mann-Whitney U-test^2^, Bonferroni correction.

**Fig 6:**
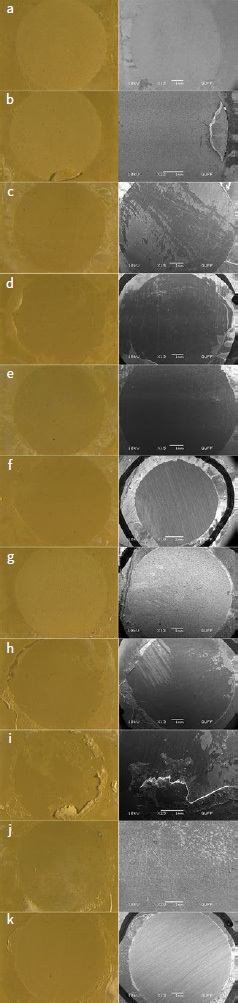
Light microscope (left) and SEM images (right) of different ARI scores. a: STC+group, ARI score 0; b: STC+ group, ARI score 1; c: ATC+ group, ARI score 0; d: ATC+ group, ARI score 1; e: SATC+ group, ARI score 0; f: CTC+ group, ARI score 0; g: STC- group, ARI score 0; h: ATC- group, ARI score 0; i: ATC- group, ARI score 1; j: SATC- group, ARI score 0; k: CTC- group, ARI score 0.

Failure modes of test groups after shear bond strength testing are given in Table 8 according to ARI. In the STC+ group (n = 10), an ARI score of 0 was determined for seven specimens, and an ARI score of 1 for three of them. In the ATC+ group (n = 10), nine specimens had an ARI score of 0, and one an ARI score of 1. Similarly, in the ATC-group (n = 10), nine specimens had an ARI score of 0, and one an ARI score of 1. All of the test specimens in the SATC+ (n = 10), CTC+ (n = 10), STC- (n = 10), SATC- (n = 10), and CTC- (n = 10) groups received an ARI score of 0. Light microscopic and SEM images of different ARI scores are shown in Fig 6.

**Table 8:** Failure mode scoring and percentages after shear bond strength testing

Adhesive remnant index	0		1		2		3	
	n	%	n	%	n	%	n	%
STC+	7	70.0	3	30.0	0	0.0	0	0.0
ATC+	9	90.0	1	10.0	0	0.0	0	0.0
SATC+	10	100.0	0	0.0	0	0.0	0	0.0
CTC+	10	100.0	0	0.0	0	0.0	0	0.0
STC-	10	100.0	0	0.0	0	0.0	0	0.0
ATC-	9	90.0	1	10.0	0	0.0	0	0.0
SATC-	10	100.0	0	0.0	0	0.0	0	0.0
CTC-	10	100.0	0	0.0	0	0.0	0	0.0


## DISCUSSION

This study showed that all applied surface treatments (sandblasting, acid etching, and sandblasting + acid etching) statistically significantly (p = 0.005) increased the roughness values of PEKK specimens. Therefore, the first hypothesis was accepted.

Previous studies reported that Al_2_O_3_ sandblasting surface treatment of PEKK changed the surface properties, created microretentive areas, and thus improved the bond strength of PEKK to resin-based materials.^26,28,42^ In studies which roughened PEKK surfaces by sandblasting with Al_2_O_3_, 50- or 110-μm particles were applied at different pressures (0.2, 0.5, 0.25, and 0.3 MPa, ie, 2, 5, 2.5, and 3 bars) from different distances (1, 5, 10, and 15 mm) for different durations (5, 10, 15, and 20 s).^14,15,26,28,42,48^ Sandblasting surface treatment has been widely used as a surface roughening process in many studies on PEKK materials. For this reason, in the present study, a surface treatment consisting of sandblasting with 110-μm Al_2_O_3_ from a distance of 10 mm for 60 s at 2 bars of pressure was preferred to roughen the PEKK surface.

In a study assessing the efficacy of surface treatments on the surface roughness of PEKK and the bond strength to composite resin veneer materials, Fokas et al^14^ reported that the group receiving sandblasting surface treatment with 110-μm Al_2_O_3_ had statistically significantly greater surface roughness than did the control group which did not receive the surface treatment; this is similar to the results of the present study.

Several studies used piranha solution (a 98% H_2_SO_4_, 30% H_2_O_2_ mixture)^17,23,35,40,46,50^ or sulfuric acid,^7,8,18,33,35,38,40,46,50,53^ which acid etches PEEK and thus roughens it. Similarly, although studies exist in which the PEKK surface is roughened with sulfuric acid,^14,28,36^ no study has yet been found that performed roughening with piranha solution.

In a previous study in which the PEEK surface was treated with piranha solution, it was emphasized that the application of piranha solution to the PEEK surface increases the micro-roughness of the surface as well as the number of functional groups. It has been stated that when treating the PEEK surface only with sulfuric acid, the sulfuric acid attacks only carbonyl and ether groups. When piranha solution is applied, the hydrogen peroxide reacts with sulfuric acid, releasing atomic oxygen, which reacts with the benzene ring. This reaction causes oxidation of the PEEK polymer, increases the polarity of the surface, and opens the aromatic ring. Thus, a greater number of functional groups that can react with the adhesive are released. It has also been stated that applying piranha solution together with Al_2_O_3_ sandblasting would provide a synergistic effect and positively influence the bond strength of resin materials to PEEK.^17^ In our study, some PEKK specimens were roughened with piranha solution. Another surface treatment group was sandblasted with Al_2_O_3_, and then piranha solution was applied.

Keul et al^23^ and Rosentritt et al^35^ reported that treating surfaces with piranha solution did not statistically significantly change the surface roughness values of PEEK specimens. In contrast, in the present study, the roughness values of the specimens acid etched with piranha solution (group A) were significantly higher than the surface roughness values of the control group (group C) (p < 0.001). While the piranha solution used in our study contained H_2_SO_4_ (98%) and H_2_O_2_ (30%) at a 10:3 ratio, the piranha solution used in the aforementioned studies^23,35^ contained H_2_SO_4_ (98%) and H_2_O_2_ (30%) at a 1:1 ratio. In our study, piranha solution was applied for 30 s, similar to these studies.^23,35^ Therefore, it is thought that this difference is due to the different composition of the piranha solution used in our study and the structural difference of PEKK.

Keul et al^23^ stated that sandblasting surface treatment increased PEEK surface roughness to a statistically significantly greater extent than did acid etching. Similarly, the surface roughness values of the sandblasted specimens (1.73 ± 0.07 μm; group S) were statistically significantly higher (p < 0.001) than in group A (0.42 ± 0.06 μm) specimens (acid etched). This suggests that sandblasting roughens PAEK surfaces more than piranha solution does. In the present study, the SEM images showed pitted, cracked surfaces in group S, that is, rougher surfaces than the honeycomb-like microporous surfaces observed in group A (Fig 5). Otherwise, while Keul et al^23^ did not find a statistically significant roughness differences between Al_2_O_3_-sandblasted PEEK surfaces and sandblasted + acid-etched PEEK surfaces, in this study, the surface roughness values of the sandblasted specimens (group S) (1.73 ± 0.07 μm) were statistically significantly higher (p < 0.001) than specimens treated with sandblasting + acid etching (group SA) (0.82 ± 0.05 μm). This difference is thought to be due to the structural differences in the PEKK material used in our study compared to PEEK. This is backed up by our SEM images, which showed group-S PEKK to have an irregular, cracked surface with pits and protruberances, and polygonal Al_2_O_3_ particles embedded in it. In group SA, a porous surface with small cracks was observed, but the indentations observed in group S were not evident (Fig 5). This suggests that the surface roughness created by the sandblasting surface treatment decreases after the application of the piranha solution, accompanied by the disappearance of the indentations.

Some studies have supported the idea that high-performance polymer (PEEK and PEKK) dental prostheses should be roughened with a mechanical and/or chemical surface treatment, and an adhesive agent should be applied to the surface in addition to these surface treatments to form a suitable and durable bond between high-performance polymer and resin-based materials (composite resin and resin cement).^8,16,44,46^ Fuhrmann et al^15^ emphasized that the application of methyl methacrylate (MMA)-containing adhesives to PEKK surfaces – just as for PEEK – is a prerequisite for obtaining a strong and durable bond in PEKK–resin-cement bonding. They stated that multifunctional methacrylates facilitated the infiltration of the adhesive into the PEKK surface, which provided durable micromechanical locking and chemical bonding. Similarly, in our study, we applied a thin layer of an MMA/diphenyl phosphine oxide-containing adhesive (Pekk Bond) on the surface of the PEKK specimens; then, PEKK-ceramic bonding was achieved using resin cement.

In similar studies,^5,8,28,39^ bond strength tests were performed using Teflon tape in order to obtain a standard bonding thickness. In our study, following similar studies, PEKK adhesion to ceramic was achieved using resin cement and Teflon tape. Pagniano et al^31^ used a microscope glass coated with a polyethylene film to standardize the resin cement thickness, evaluating the efficacy of resin cements in terms of the long-term success of glass-ceramic crowns. As previously suggested by Jacques et al,^20^ a 0.94 kg (9.2 N) weight was applied to create the ideal resin-cement film thickness (100 µm) during the cementation of all-ceramic crowns. Similarly, in our study, each specimen was subjected to a standard pressure of 0.94 kg via a loading device to create a standard and homogeneous resin cement thickness.

Shear and tensile bond strength tests are used as in-vitro methods for determining the bonding efficacy of dental materials.^35,54^ The shear bond strength test has been utilized in various studies^26,28,35,36^ on PAEK materials. In the present study, the shear bond strength test was chosen to measure the bond strength between PEKK and ceramic, as it is widely supported by the literature and promotes comparability with our study results. Aging via thermocycling is a commonly used method for evaluating the influence of simulated intraoral conditions on the bond strength of dental materials. It has also been used in many studies on PEKK,^14,15,26,36,48,52^ including ours, performed before shear bond strength tesing in half the bonded PEKK-ceramic specimens (n = 10) from each surface treatment group. During thermocycling, the other specimens were kept in an incubator at 37°C in distilled water.

The present results showed that the shear bond strength in the thermocycled groups (TC+) was not statistically significantly affected by surface treatments (sandblasting, acid etching, and sandblasting + acid etching). In the non-thermocycled groups (TC-), sandblasting statistically significantly increased the shear bond strength (p = 0.001). Hence, the hypothesis that surface treatments would affect the bond strength of PEKK to ceramics was partially rejected. Statistically significantly lower shear bond strength was obtained in the thermocycled subgroup of the sandblasting treatment group (STC+) (11.02 ± 3.25 MPa) vs the respective non-thermocycled subgroup (STC-) (13.53 ± 1.84 MPa) (p = 0.015). However, statistically significantly higher shear bond strengths resulted in the thermocycled subgroup of the sandblasting + acid etching group (SATC+) (12.3 ± 3.1 MPa) vs the respective non-thermocycled subgroup (SATC-) (8.67 ± 2.43 MPa) (p = 0.011). Thermocycling did not create a statistically significant difference in the acid etching surface treatment group (group A) (p = 0.393) or the control group (group C) (p = 0.853). Thus, the hypothesis that thermocycling would have a negative effect on bond strength was partially rejected.

The thermal stress created by thermocycling accelerates the diffusion of water at the interface of the bonded materials and causes the materials to expand and contract.^36^ Thermocycling may cause mechanical stress on the bonding area resulting from different volumetric changes of the materials and therefore tends to decrease the bond strength; it may also increase the bond strength as a result of post-polymerization of the adhesive and resin-based materials.^32,47^ Labriaga et al^26^ reported that thermocycling statistically significantly reduced the shear bond strength of resin cement to PEKK without surface treatment (only an MMA-containing adhesive was applied). Sakihara et al^36^ stated that thermocycling reduces the shear bond strength of composite resin to PEKK without surface treatment (only a 10-MDP–containing adhesive was applied); this difference was not statistically significant. The results of the present study show similarities with the study by Sakihara et al,^36^ but differences from the study by Labriaga et al.^26^ Thermocycling reduced the shear bond strength in the control group, but this was not statistically significant (p = 0.853) in our study. The resin cement (G-CEM Link Force) used here to bond PEKK to ceramic was a dual-cure self-adhesive resin cement similar to that used by Labriaga et al (RelyX Unicem).^26^ Furthermore, the adhesives used were similar, both containing MMA. The differences between our results and those of Labriaga et al might instead be attributable to the different number of thermal cycles employed: 6000 in the current study, and 10,000 cycles in the Labriaga et al study.^26^ Those authors stated that thermocycling reduced the shear bond strength of test specimens treated with sandblasting, in agreement with our results.

In the current study, in the acid-etching surface treatment group (group A), thermocycling non-significantly decreased shear bond strength (p = 0.393). In the sandblasting + acid etching surface treatment group (group SA), thermocycling significantly increased the shear bond strength (p = 0.011). Although the same adhesive agent and resin cement were applied in all surface treatment groups, thermocycling decreased the shear bond strength in some surface treatment groups (S, A, and C), but only in group S was this statistically significant (p = 0.015). Moreover, the shear bond strength in group SA (p = 0.011) increased significantly. More in-vitro studies are needed to verify these results

Labriaga et al^26^ assessed the shear bond strength of PEKK specimens with resin cement, applying various treatments to the PEKK surface. Half of the specimens were thermocycled, while the other half were not. With thermocycling, the bond strengths of the sandblasting group (12.1 ± 4.6 MPa) roughened with 50-μm Al_2_O_3_ were found to be statistically significantly higher compared to the control group (4.8 ± 3.7 MPa) without surface treatment. Similarly, the present study showed that the bond strengths in the thermocycled subgroup of the sandblasting group (STC+) (11.02 ± 3.25 MPa) were greater than in the control group (CTC+) (8.41 ± 4.01 MPa), but this difference was not statistically significant (p = 0.393). Our study observed that the shear bond strength of the CTC+ group (8.41 ± 4.01 MPa) was greater than that of the control group (4.8 ± 3.7 MPa) in the study by Labriaga et al.^26^ Although no surface treatment was applied, this difference is thought to be due to the difference in the adhesive agent applied after the surface treatments in our study. As recommended by the PEKK manufacturer, the present study used Pekk Bond (MMA, diphenyl phosphine oxide-containing adhesive agent); Labriaga et al^26^ used Visio.link (Senden, Germany; MMA, dimethacrylate, and pentaerythritol triacrylate [PETIA]). Those authors found that the bond strength of the sandblasting group was statistically significantly greater than that of the control group (no surface treatment) in test specimens without thermocycling. Similarly, we found that in the non-thermocycled subgroup, the bond strength of the sandblasting group (STC-) (13.53 ± 1.84 MPa) was statistically significantly (p = 0.001) higher than that of the control group (CTC-) (9.14 ± 2.53 MPa).

The higher shear bond strengths mediated by sandblasting suggest that this surface treatment has a positive effect on the bonding of PEKK materials with resin cements. The SEM images and surface roughness data obtained in the present study also support this idea. When the SEM images of PEKK specimens treated with sandblasting (group S) were compared with the untreated PEKK specimens (control group), rougher and more indented surface features were detected. It is thought that the undercuts formed as a result of sandblasting contribute positively to the bond strength. The surface roughness data obtained in the present study also show that sandblasting increased the roughness of the PEKK specimens. This supports the idea that the bond strength will also increase with increasing surface roughness. It reveals that sandblasting is an effective surface treatment for enhancing the bond strength of PEKK to ceramics bonded with resin cement.

Studies on piranha solution surface treatment showed that it removes organic residues, increases surface polarity, breaks aromatic structures, and considerably increases bond strength between PEEK and resin-based materials.^17,46^ Silthampitag et al^40^ investigated the shear bond strength of PEEK materials with composite resins without thermocycling by applying various treatments to the PEEK surface. They found that the shear bond strength increased significantly between composite resin and PEEK with piranha solution surface treatment. We found that the shear bond strength of the non-thermocycled subgroup of acid etching with piranha solution (ATC-) (11.86 ± 2.83 MPa) was non-significantly (p = 0.019) higher than that of the control group (CTC-) (9.14 ± 2.53 MPa). The present results documented higher bond strength of the non-thermocycled control (CTC-) specimens (9.14 ± 2.53 MPa) compared to the control group (1.14 ± 0.72 MPa) of Silthampitag et al.^40^ We propose that this was due to the fact that the adhesive agent (Heliobond) used by Silthampitag et al^40^ contained bis-GMA and TEG-DMA, unlike our study.

The adhesive agent containing MMA (Pekk Bond) used in our study provided effective bonding even in the control group, to which no surface treatment was applied. Silthampitag et al^40^ reported statistically significantly higher bond strengths of PEEK specimens to which piranha solution had been applied, compared to sandblasted PEEK specimens. In contrast, the present study found no statistically significant difference (p = 0.436) between the shear bond strength of the non-thermocycled sandblasted (STC-) specimens (13.53 ± 1.84 MPa) and those of non-thermocycled acid-etched (piranha solution) specimens (group ATC-) (11.86 ± 2.83 MPa). It is possible that the surface roughness produced by sandblasting with 110-μm Al_2_O_3_ in our study – instead of sandblasting with 50-μm Al_2_O_3_ as Silthampitag et al^40^ did – yields higher bond strengths.

Our non-thermocycled sandblasted subgroup (STC-) (13.53 ± 1.84 MPa) revealed higher shear bond strength than did the sandblasting group (5.60 ± 2.26 MPa) in Silthampitag et al.^40^ In the present study, the roughness values of specimens treated with sandblasting (group S) (1.73 ± 0.07 µm) were higher than those of Silthampitag et al^40^ (0.37 ± 0.05 µm). This suggests that in our study, a more effective sandblasting procedure was applied, and the shear bond strength was positively affected by increasing surface roughness. Unlike the adhesive agent used by Silthampitag et al,^40^ our use of an MMA-containing adhesive agent may also be one of the factors that positively affects shear bond strength.

Rosentritt et al^35^ determined that the shear bond strengths of piranha-solution-treated PEEK specimens to composite resin – without applying a bonding agent – were 0.0 MPa in the absence of aging. In contrast, the present shear bond strength (11.86 ± 2.83 MPa) of the non-thermocycled acid-etched (piranha solution) subgroup (ATC-) of PEKK was >10 MPa, a value considered clinically acceptable.^35^ This difference is thought to be due to application of an adhesive agent to the PEKK surface in our study. Rosentritt et al^35^ stated that, for effective bonding, surface roughening is essential before using an adhesive agent; however, they emphasized that surface roughening alone is not effective. We observed that using an MMA-containing adhesive agent (Pekk Bond) ensured higher shear bond strengths in the non-thermocycled control group (CTC-) (9.14 ± 2.53 MPa) vs the group in which Rosentritt et al^35^ applied piranha solution as surface treatment. This observation, as discussed in the literature,^15,22,47^ reveals that MMA-containing adhesive agents positively influence the bonding of PEKK with resin-based materials.

A previous study^17^ stated that the tensile bond strength between resin-based materials and PEEK surfaces increased with application of piranha solution to the PEEK surface, together with Al_2_O_3_ sandblasting and an adhesive agent. However, these data were obtained without thermocycling. In the current study, in terms of the shear bond strengths of the non-thermocycled subgroups of surface treatment groups (STC-, ATC-, SATC-, and CTC-), the sandblasting group (STC-) showed statistically significantly higher (p < 0.001) bond strength than the sandblasting + acid etching group (SATC-). Our study also showed that the shear bond strength of the acid etching (piranha solution) group (ATC-) was significantly higher than the sandblasting + acid etching group (SATC-) (p = 0.003). The use of acid etching (piranha solution) together with sandblasting did not provide a synergistic effect in our study. This is supported by our SEM images, which showed that the indented and protruding, irregular, sharp surfaces produced by sandblasting were subsequently flattened as a result of etching; specifically, the edges were rounded and a smoother porous surface was formed. Considering the surface roughness data in our study, the decreased roughness of the sandblasted surface due to acid etching – illustrated by smoother, rounded surfaces in the SEM images – suggests that the use of piranha solution together with sandblasting does not provide a synergistic effect in terms of bond strength. The test specimens treated with acid etching (group A) showed statistically significantly (p < 0.001) lower surface roughness values than the test specimens treated with sandblasting + acid etching (group SA). However, shear bond strengths were statistically significantly (p = 0.003) higher in the non-thermocycled acid-etched subgroup (ATC-) than in the non-thermocycled sandblasted + acid etched (SATC-). However, instead of the regular, honeycomb-like structure seen in SEM images of test specimens treated with acid etching (group A), smoother and rounded porous surfaces were observed in specimens treated with sandblasting + acid etching (group SA). This suggests that piranha solution only causes the PEKK surface to dissolve enough to smooth the sharp edges of the sandblasted surface, and is not sufficiently effective after sandblasting.

Keul et al^23^ examined the tensile bond strength between PEEK and composite veneers by soaking the specimens in distilled water at 37°C for 60 days, and then subjecting the specimens to 5000 thermal cycles. They reported that both PEEK test groups treated with sandblasting and sandblasting + acid etching (piranha solution) had statistically significantly higher tensile bond strengths compared to the untreated control group or the group treated only with acid etching (piranha solution). No statistically significant difference was found between the control group and the piranha solution group, or between the sandblasting group and the sandblasting + acid etching (piranha solution) group in terms of tensile bond strength. Similarly, in our study, both thermocycled specimens first treated with sandblasting (STC+) and thermocyled specimens first treated with sandblasting + acid etching (SATC+) had higher shear bond strengths compared to the specimens with no surface treatment (CTC+) or with acid etching alone (ATC+). However, in our study, the differences in shear bond strength among the thermocycled surface treatment groups (STC+, ATC+, SATC+, and CTC+) were not significant. While both studies mentioned above reported tensile bond strengths between PEEK and composite resins,^17,23^ our study discusses shear bond strengths.

For the evaluation of bonding success, it is necessary to examine the type of separation that occurs at the bonding interface as well as the results of the bond strength test. In similar studies on the bond strength of PEKK to resin cement or composite-resin veneering material, the types of failures are classified as adhesive, cohesive, and mixed.^14,15,26,28,42,52^ Although the most common type of adhesive failure was observed between PEKK and the composite material in one study,^14^ it was stated that more studies were needed to understand the failure mechanism.^14^ However, while other bond strength studies related to PEKK only evaluated the bond to resin cement or composite veneering material,^15,26,28,42,52^ the bonding of PEKK to ceramic materials with resin cement was investigated in our study. Årtun and Bergland,^1^ on the other hand, bonded orthodontic brackets with adhesive resin to teeth that underwent various surface treatments, and gave scores of 0, 1, 2, or 3 using the ARI (adhesive remnant index), according to the amount of adhesive remaining on the tooth surface after bracket removal. In many similar studies examining the shear bond strength of ceramic or metal orthodontic brackets mediated by adhesive resin to ceramic or zirconia crowns,^19,29^ bond failure was evaluated with the ARI of Årtun and Bergland.^1^ In our study, the ARI was used instead of the failure-type classification (which classifies failures as adhesive, cohesive, and mixed) because we examined the shear bond strength of PEKK not only to resin cement but also to ceramic using resin cement. The dominant score in all test groups in our study was 0. The fact that only 0 and 1 scores were given in our study indicates that all of the resin cement (score 0), or the majority (score 1), remained on the lithium-disilicate glass-ceramic at the interface of PEKK and lithium-disilicate glass-ceramic after bonding failure. This shows that the resin cement used in our study bonds better to the lithium-disilicate glass-ceramic material than the PEKK material does. Although there is no study examining the bond strength between PEKK and ceramics in the literature, many studies have examined the bond strength of PEKK to resin-based materials, reporting that the majority of all resin-cement/composite-resin veneering material separates from the PEKK surface.^14,15,42^ In this respect, the results are similar to our study.

ISO 10477 (2004) data indicate that the minimum acceptable bond strength between resin-based materials and the substrate is 5 MPa.^55^ In our study, the bond strengths of all surface treatment groups with and without thermocycling were higher than 5 MPa, and were thus deemed acceptable. Data obtained immediately after bonding with resin-based materials only provide early bond strengths; however, bond strength after aging (with thermocycling or long-term storage) can provide a prediction about longer-term performance in-vivo.^34,35^ In this context, the literature mentions that only shear bond strengths >10 MPa are acceptable for clinical use.^2,4^ By this definition, as the shear bond strengths of groups STC+ (11.02 ± 3.25 MPa), ATC+ (10.86 ± 2.95 MPa), and SATC+ (12.3 ± 3.1 MPa) were >10 MPa, they would be acceptable for clinical use. The results of our study show that sandblasting, acid etching, and sandblasting + acid etching of PEKK surfaces play an important role in the bonding of PEKK to lithium-disilicate glass-ceramic. Our test results show that the absence of surface treatment could not create the necessary bond of PEKK and to lithium-disilicate glass-ceramic. To improve the effectiveness of the bond strength of PEKK to ceramics, it is recommended to consider diverse surface treatments; in-vitro studies are needed on this subject. At the same time, increasing the number of cycles in the thermocycling process is important in terms of predicting long-term clinical performance in prospective studies.

## CONCLUSIONS

Surface treatments (sandblasting, acid etching, and sandblasting + acid etching) created statistically significantly degrees of roughness on the PEKK surfaces. While specimens treated with sandblasting displayed the highest roughness values, specimens treated with the acid etching demonstrated the lowest roughness values.

The application of sandblasting, acid etching, and sandblasting + acid etching surface treatments to PEKK played an important role in the bonding of PEKK to lithium-disilicate glass-ceramic material. To create an effective bond between the PEKK material and lithium-disilicate glass-ceramic, omitting such surface treatment in clinical practice should not be recommended.

## Clinical relevance:

The roughening of the PEKK surface with surface treatments and the application of adhesive agents improve the bond strength to ceramic.
